# Immunomodulation: Immunoglobulin Preparations Suppress Hyperinflammation in a COVID-19 Model *via* FcγRIIA and FcαRI

**DOI:** 10.3389/fimmu.2021.700429

**Published:** 2021-06-10

**Authors:** Fabian Bohländer, Dennis Riehl, Sabrina Weißmüller, Marcus Gutscher, Jörg Schüttrumpf, Stefanie Faust

**Affiliations:** ^1^ Department of Analytical Development and Validation, Corporate R&D, Biotest AG, Dreieich, Germany; ^2^ Corporate R&D, Biotest AG, Dreieich, Germany; ^3^ Department of Translational Research, Preclinical Research, Corporate R&D, Biotest AG, Dreieich, Germany

**Keywords:** SARS-CoV-2, COVID-19, IVIG, trimodulin, ITAMi, Fc-receptors, immune modulation, neutrophils

## Abstract

The rapid spread of SARS-CoV-2 has induced a global pandemic. Severe forms of COVID-19 are characterized by dysregulated immune response and “cytokine storm”. The role of IgG and IgM antibodies in COVID-19 pathology is reasonably well studied, whereas IgA is neglected. To improve clinical outcome of patients, immune modulatory drugs appear to be beneficial. Such drugs include intravenous immunoglobulin preparations, which were successfully tested in severe COVID-19 patients. Here we established a versatile *in vitro* model to study inflammatory as well as anti-inflammatory processes by therapeutic human immunoglobulins. We dissect the inflammatory activation on neutrophil-like HL60 cells, using an immune complex consisting of latex beads coated with spike protein of SARS-CoV-2 and opsonized with specific immunoglobulins from convalescent plasma. Our data clarifies the role of Fc-receptor-dependent phagocytosis *via* IgA-FcαRI and IgG-FcγR for COVID-19 disease followed by cytokine release. We show that COVID-19 associated inflammation could be reduced by addition of human immunoglobulin preparations (IVIG and trimodulin), while trimodulin elicits stronger immune modulation by more powerful ITAMi signaling. Besides IgG, the IgA component of trimodulin in particular, is of functional relevance for immune modulation in this assay setup, highlighting the need to study IgA mediated immune response.

## Introduction

Corona virus induced disease 2019 (COVID-19) is a global threat induced by the rapid spread of the newly emerged severe acute respiratory syndrome coronavirus 2 (SARS-CoV-2). Although most infected individuals recovered rapidly, approximately 20% of patients with COVID-19 pneumonia cannot clear the virus and develop severe COVID-19 (1, 2). These patients are characterized by an exhausted immune system with hyperinflammation related severe acute respiratory distress syndrome (ARDS) and so called “cytokine storm” (3, 4). COVID-19 relevant inflammatory cytokines like interleukin (IL)-6, IL-8, tumor necrosis factor-α (TNF-α), monocyte chemoattractant protein-1 (MCP-1) or IL-1β, and anti-inflammatory cytokines like IL-10 or IL-1 receptor antagonist (IL-1ra) were shown to correlate with disease severity (5–8). Previous data indicate that excessive immune response by the host is of greater importance for COVID-19 progression than virus induced damage alone (9). Therefore, downmodulation of hyperinflammation in severe COVID-19 patients is of major importance (4, 10–13). Available immunomodulatory drugs are intravenous immunoglobulin preparations (IVIGs) (10). IVIGs have been used for treatment of inflammatory diseases for decades, and first clinical studies have showed promising results in treatment of COVID-19 patients (14–17). Furthermore IgA- and IgM-enriched immunoglobulins are available or are in clinical testing (18, 19). These immunoglobulin therapeutics are known to have benefits in other inflammatory diseases (20–23); however, whether they are advantageous in COVID-19, has yet to be evaluated.

Understanding the mode of action of these drugs is essential to estimate clinical success. To achieve this, appropriate model systems for virus and human immunology are necessary (24). *In vivo* models, mainly mice, can mimic human immunity quite well, but in the case of severe COVID-19, critical limitations remain. Another approach is an *in vitro* model with virus-like particles based on beads coated with surface protein of the virus. This technique has so far been used to measure neutralizing antibody levels against common viruses (25, 26).

Antibodies are a versatile and important component of our immune system. Antibodies mediate effector functions *via* their Fc-region and corresponding Fc-receptors (FcRs) to defeat invading pathogens (27). In addition, in COVID-19, antibody mediated effector functions are crucial for disease outcome and severity (8, 26, 28–30). On the other hand, FcRs are important regulators of immunity (31, 32). FcR-dependent immune modulation by IVIG is assumed to be beneficial in hyperinflammatory COVID-19 patients, but there is currently no experimental evidence (33, 34).

Another key player in COVID-19 disease is neutrophil infiltration (neutrophilia) which was shown to be associated with poor clinical outcomes of severe cases (35). Previous data indicate a detrimental role of activated neutrophils resulting in tissue damage (35).

Considering the discussed challenges regarding hyperinflammation, the lack of appropriate model systems, the modulatory role of immunoglobulin preparations and neutrophilia, a new COVID-19 model is needed. The goal of our work was the establishment of a platform technology which enables investigations regarding inflammation as well as immunomodulation by immunoglobulin preparations under standard laboratory conditions. In our neutrophil *in vitro* system we can induce hyperinflammation comparable to severe COVID-19 and modulate cytokine release by adding classical IVIG (~98% IgG, <2% IgA/IgM) as well as IgA and IgM containing trimodulin. Trimodulin is a normal polyvalent antibody preparation derived from plasma for intravenous administration. Trimodulin contains immunoglobulins IgM (~23%), IgA (~21%), and IgG (~56%). Our model enables the detailed investigation of neutralizing IgG, IgM, and IgA as well as FcγR and FcαRI-dependent immune modulation by IgG and the so far neglected IgA *via* inhibitory immunoreceptor tyrosine-based activation motif (ITAMi) signaling.

## Materials and Methods

### Plasma Samples

COVID-19 plasma was collected after PCR confirmed SARS-CoV-2 positive test. Anonymized donors were fully recovered and tested negative for SARS-CoV-2 RNA. Plasma collection was performed *via* plasmapheresis at Plasma Service Europe. Donations were stored at −70°C for long-term. Donations were pre-selected for IgG, IgA, and IgM positive antibodies *via* commercial test-kits (Virotech SARS-CoV-2 IgG/A/M ELISA); in total 86 donations were screened. Donors agreed with ethical consent form for the use of their donation in research.

### Cell Line and Culture Conditions

HL60 cells (ATCC #CCL-240) were cultured in Iscove Modified Dulbecco Media (IMDM) (Life technologies) supplemented with 20% heat-inactivated fetal bovine serum (FBS) (Life technologies) and 1% Penicillin/Streptomycin (Sigma-Aldrich) at 37°C and 5% CO_2_. To induce neutrophil-like phenotype, HL60 cells were centrifuged (350 × g, 5 min) and resuspended in supplemented IMDM with 1.3% (v/v) Dimethylsulfoxide (DMSO) (Sigma-Aldrich) to 6 × 10^5^ cells/ml and incubated for 4 days at 37°C (36). Cell phenotype was confirmed by flow cytometry analysis. For further investigations, primary human neutrophils were isolated from human blood (see **Supplementary Methods**).

### Flow Cytometry Analysis

To ensure differentiation of HL60 cells to a neutrophil-like phenotype well known surface markers CD71, CD35, CD15, CD193, and CD11b were compared with primary human neutrophils. HL60 cells were washed twice with phosphate buffered saline (PBS) and then pre-incubated with human IgG (IVIG) (*IgG Next Generation*, Biotest AG) to block non-specific binding (37). Fluorophore labeled detection antibodies for FcR, surface markers, and corresponding isotype controls were purchased from companies listed in **Table SI**. Further viability stain Zombie Aqua (BioLegend) was added to staining mix. To stain 1 × 10^6^ cells’ multicolor antibody mixes were prepared. FcγRIIB clone 2B6 was labeled with AF647 NHS-Ester (Thermo Fisher Scientific) according to the manufacturer’s instructions. Staining was performed for 30 min at 4°C in the dark. Cells were analyzed using FACS Canto II Cytometer (BD Biosciences), and gating was performed according to **Figure S1**. In more detail, FcR expression was quantified as described in **Supplementary Material**.

### Coating of Beads

Yellow-green fluorescent latex beads (1 µM diameter) (Sigma-Aldrich) were washed twice with a buffer consisting of 50 mM 2-(N-Morpholino)ethanesulfonic-acid (MES) (Sigma-Aldrich) and 1.3 mM N-Ethyl-N′-(3-dimethylaminopropyl)-carbodiimide-hydrochloride (EDAC) (Sigma-Aldrich) pH 6.1. Covalent coating of latex beads was performed by adding reSARS-CoV-2 full-length spike protein His-tag (Acro Biosystems) to beads in MES/EDAC-Buffer. The mixture was incubated for 2 h at 37°C. Thereafter 2% bovine serum albumin (BSA) (Sigma-Aldrich) in PBS was added for blocking.

### Preparation of SARS-CoV-2-Like Immune Complex

A 5 µg/ml chimeric mouse/human IgG1 specific to SARS-CoV-2 receptor binding domain antibody (Sino Biological #40150-D001-50) or 400 µg/ml heat inactivated convalescent COVID-19 plasma (Biotest AG) was added to the coated beads. Immune complex (IC) formation was performed by incubating SARS-CoV-2 specific antibodies (recombinant or plasma source) with coated beads for 45 min at 37°C. As controls, immunoglobulins (negative against SARS-CoV-2 spike protein) or BSA was mixed with coated beads and incubated as described.

### Characterization of Immune Complex

Additional analysis of coated and opsonized beads was performed by detecting IgG, IgA, and IgM on the surface of beads by flow cytometry. Staining and measurement procedures were performed as described above in flow cytometry analysis. Centrifugation steps were performed with 4,700 × g for 15 min. The following detection antibodies were used: anti-human IgA-VioBlue (Miltenyi Biotec), anti-human IgM-PE-Cy5, and anti-human IgG-APC-H7 (both BD Biosciences). Isotype controls were used to set gates as shown in **Figure S2**.

### Phagocytosis Assay

Neutrophil-like HL60 cells were resuspended at 1.25 × 10^6^ cells/mL in IMDM without supplements. Immune complex was washed with PBS. After resuspension in IMDM without fetal bovine serum FBS, SARS-CoV-2 immune complex was added to the cells and incubated for phagocytosis 1 h at 37°C. After incubation, cells were centrifuged (350 × g, 5 min), and supernatant was stored for cytokine measurements at −20°C. Pellets were washed twice and incubated with 0.2% trypane-blue (Gibco) solution to quench extracellular fluorescence. Fluorescent bead uptake was monitored on FACS-Canto II cytometer. Evaluation and gating are shown in **Figure S3**. Phagocytic index was calculated by multiplying percentage of positive cells by the median fluorescence intensity of positive cells.

### FcR Blocking Experiments

For blocking experiments 5 µg/ml of the following antibodies was used: anti-human FcαRI, clone MIP8α (Bio-Rad); anti-human FcγRI, clone 10.1 (BioLegend); anti-human FcγRIIA, clone IV.3 (StemCell); anti-human FcγRIIB, clone 2B6 (Creative BioLabs); anti-human FcγRIII, clone 3G8 (BioLegend). Cells were pre-incubated with blocking antibodies for 20 min before adding the SARS-CoV-2-like immune complex to the cells. The phagocytic index of non-blocked cells was referenced as 100%, and the percentage of remaining signal was calculated.

### Immune Modulation by IVIG or Trimodulin

To show immune modulatory effects of classical IVIG (*IgG Next Generation, Biotest AG*) and IgA and IgM enriched immunoglobulin (*trimodulin, Biotest AG*), SARS-CoV-2-like immune complexes were prepared, and the phagocytosis assay was performed as described. Following addition of immunoglobulin opsonized immune complex 0.005–15 g/L, IVIG or trimodulin was added to the cell–bead mixture. Cells were incubated for 1 h and analyzed by flow cytometry as described above.

### ITAMi Signaling Experiments

To test ITAMi signaling as mechanism of immunoglobulin induced immune modulation, SHP-1 phosphatase was inhibited using NSC-87877 inhibitor (38), and SHP-1 phosphorylation was analyzed at tyrosine 536 (pY536) (39). IL-8 release into cell culture supernatant was compared between inhibitor addition or not. Therefore, cells were pre-incubated with SHP-1 inhibitor for 30 min before phagocytosis assay. Further, to directly show SHP-1 activation by immunoglobulin induced immune modulation. Cells were treated for 90 min with SARS-CoV-2-like immune complex, trimodulin or IVIG. Then cells were fixed, permeabilized, and stained with FITC conjugated anti-SHP-1-pY536 specific antibody (Abwiz Bio Inc.). The fluorescence signal of immune complex treated cells was normalized to 100%, and percentage change due to trimodulin or IVIG addition was calculated.

### Measurement of Cytokine Release

Quantification of cytokine levels in cell culture supernatants was performed using human IL-10, IL-8, IL1ra, MCP-1, MIP-1*α* simple step ELISA Kit (Abcam). The assays were performed according to the manufacturer’s instructions. For qualitative comparison of secreted cytokines, a human cytokine array kit (R&D systems) was used. The assay was performed as described by the manufacturer, and membranes were incubated with 1:2,000 diluted IRDye-800CW Streptavidin (LI-COR) and stained for 30 min. Images were collected using an Odyssey-Infrared-Imaging System, and spot intensities were analyzed using the grid-array function.

### Statistical Analysis

All data are expressed as mean ± standard deviation of the indicated number of measurements. Statistics were calculated using GraphPad Prism 6.1 Software. Two-way analysis of variance (ANOVA) with Tukey’s multiple comparison test or one way ANOVA with Dunnett’s multiple comparison test was performed as indicated. Significance was quantified as p-values with asterisks: *p ≤ 0.05, **p ≤ 0.01, ***p ≤ 0.001, ****p ≤ 0.0001; with 95% confidence interval.

## Results

### Preparation of SARS-CoV-2-Like Immune Complexes

**Figure 1:**
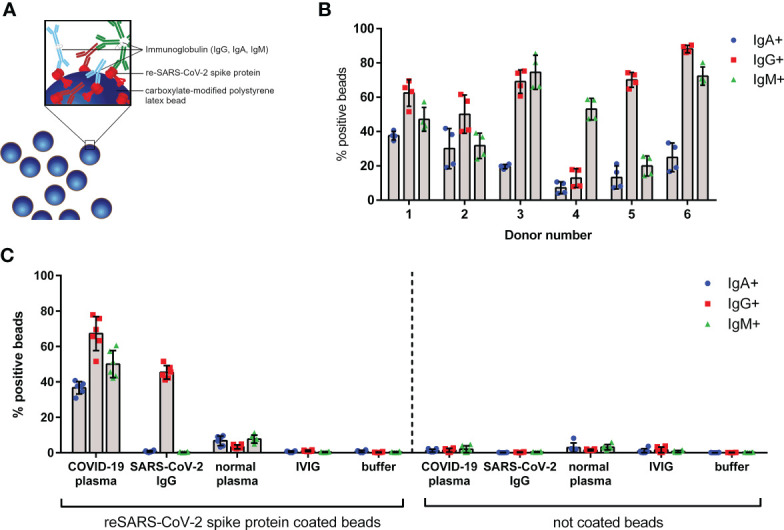
Characterization of SARS-CoV-2-like particles. **(A)** Schematic overview of fluorescent latex beads coated with recombinant (re-) SARS-CoV-2 spike protein; specific immunoglobulins of IgG (red), IgA (blue), and IgM (green) classes bound to the spike protein are shown. **(B)** IgG, IgA, and IgM bound on surface of SARS-CoV-2-like latex beads opsonized with different convalescent COVID-19 plasma donations (donor numbers 1–6). Coated beads were incubated with plasma from indicated donors for 45 min at 37°C. After washing, beads were stained with anti-IgG, anti-IgA, and anti-IgM detection antibodies and analyzed by flow cytometry. Percentage of positive beads for IgG, IgA, and IgM is shown; data represents mean of four independent experiments. **(C)** Control experiments show specific binding of anti-SARS-CoV-2 antibodies to spike protein. Coated or non-coated beads were incubated with indicated plasma, immunoglobulins, or buffer for 45 min at 37°C and were stained as described in **(B)**. Data represents mean of six independent experiments.

Establishing a COVID-19 inflammation model requires a solution that is both simple and workable under standard laboratory conditions as a feasible alternative to native SARS-CoV-2 virus. As basis for our SARS-CoV-2-like immune complex we therefore used fluorescent latex beads. We coated the beads with recombinant SARS-CoV-2 spike protein to generate SARS-CoV-2-like particles. Immune complex generation was performed by adding SARS-CoV-2 spike protein specific IgG, IgA, and IgM antibodies from convalescent COVID-19 source plasma (**Figure 1A**).

Plasma from six convalescent donors was characterized for binding of IgG, IgA, and IgM to the bead surface (**Figure 1B**). The data indicates that all used COVID-19 plasma donations have IgG, IgA, and IgM antibodies in various amounts against the SARS-CoV-2 spike protein. To exclude non-specific antibody binding to the beads, control experiments were performed. As depicted in **Figure 1C**, neither IgG, IgA, nor IgM was detected on SARS-CoV-2 spike protein coated beads by addition of normal plasma, IVIG, or buffer. In contrast, beads incubated with recombinant anti-SARS-CoV-2 IgG control showed 45% IgG positive beads, however, no IgA or IgM. Supporting the data from coated beads, no antibodies were detected on uncoated (but blocked) latex beads. For further experiments we used plasma donation 1 (hereafter named COVID-19 plasma), as this donation was available at sufficient quantities and exhibits an equal binding of IgG, IgA, and IgM antibodies on the SARS-CoV-2 spike protein coated beads.

### COVID-19-Like Inflammation Model

For induction of COVID-19-like inflammation the manufactured immune complex must be phagocytosed by immune effector cells *in vitro*. Based on the bead characterization data we expected antibody-FcR-dependent phagocytosis of SARS-CoV-2-like immune complex and subsequently the release of several pro-inflammatory cytokines and chemokines. Phagocytosis was measured by uptake of fluorescent beads, while cellular inflammation was detected by cytokine and chemokine release into cell culture supernatant. Neutrophil-like cells were chosen as immune effector cells.

**Figure 2:**
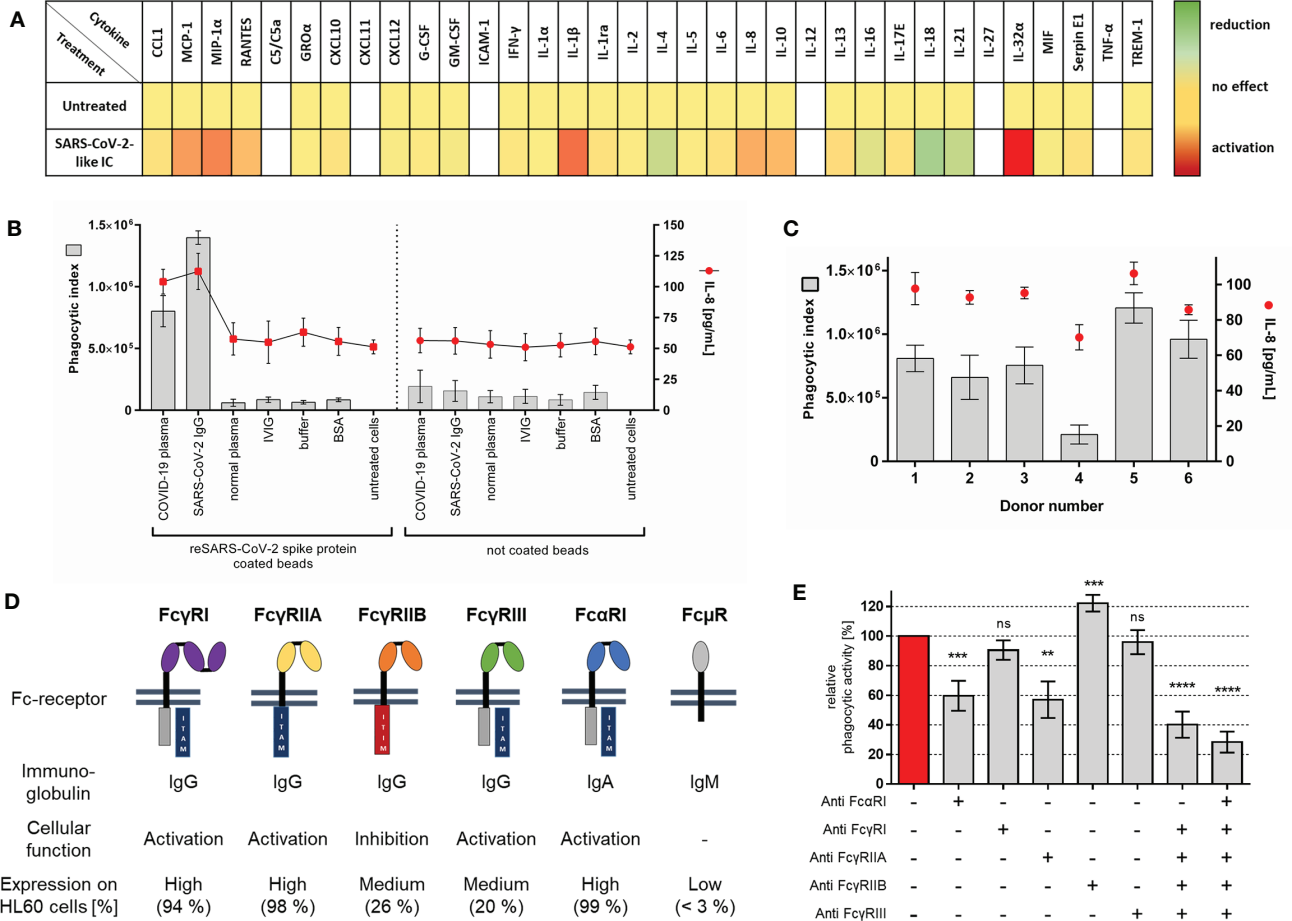
Establishment of COVID-19 like inflammation model **(A)** Heat map of chemokines and cytokines secreted by neutrophil-like HL60 cells after stimulation with anti-SARS-CoV-2 IgG-IC. HL60 cells were stimulated for 18 h at 37°C with the indicated immune complex. Qualitative cytokine secretion was measured by using human cytokine arrays. Relative signal intensities for every measured cytokine of untreated cells were set as 1 (yellow). The x-fold induction (red) or reduction (green) in comparison to untreated cells was calculated. Not detected cytokines are shown in white. Results represent mean of three independent experiments. **(B)** Evaluation of COVID-19-like inflammation model. Cells were incubated for 1 h with SARS-CoV-2 spike protein coated or not coated beads opsonized with different immunoglobulins, BSA, or PBS controls. Phagocytic index (gray bars, left y-axis) for bead uptake and corresponding IL-8 release (red dots, right y-axis) in cell culture supernatant were measured. Data represents mean of eight independent experiments. **(C)** Analysis of phagocytic activity and IL-8 release with different COVID-19 plasma donors [experimental procedure as in **(B)**]. **(D)** Overview of human Fc-receptors (FcR), immunoglobulin binding specificity, cellular function, and their expression on neutrophil-like HL60 cells. Fc-receptor expression on HL60 cells, 4 days differentiated with 1.3% DMSO, was measured as percentage of positive cells; results represent mean of three independent experiments. **(E)** FcR blocking experiments with COVID-19 plasma opsonized SARS-CoV-2-like particles. Cells were pre-incubated with 5 µg/ml of indicated blocking antibodies or combinations of those 20 min before addition of immune complex. Phagocytic index of not blocked cells was referred as 100% phagocytic activity, and the remaining phagocytic activity is shown as mean of six independent experiments. Statistics: One way ANOVA; Dunetts multiple comparisons test, 95% confidence interval. ns, not significant, **p ≤ 0.01, ***p ≤ 0.001, ****p ≤ 0.0001.

For our novel neutrophil *in vitro* system, we used the well characterized HL60 cell line and compared them before and after differentiation to a neutrophil-like phenotype with primary human neutrophils isolated from blood. The neutrophil-like phenotype including expression of SARS-CoV-2 angiotensin converting-enzyme-II (ACE-II) receptor (**Table SII** and **Figure S4A**) and FcR expression on cell surface, was investigated (**Table SII** and **Figure S4B**). During differentiation with DMSO, HL60 cells changed to a neutrophil-like cell phenotype, shown by increased expression of neutrophil markers CD11b, CD35, CD15, FcγRIII, and FcαRI (**Figure S4**). Primary human neutrophils displayed a similar phenotype (**Table SII**). High expression of activating FcγRI, FcγRIIA, and FcαRI, as well as lower extent of FcγRIIB and FcγRIII was detected on neutrophil-like HL60 cells (**Figure 2D**). Primary neutrophils in comparison showed higher levels of FcγRIII, but lower FcγRI expression (**Table SII**). FcµR and ACE-II receptor expression was minimal on neutrophil-like HL60 cells as on primary neutrophils (**Table SII** and **Figure S4**).

We tested if SARS-CoV-2-like immune complex can induce inflammation similar to that of severe COVID-19. The pattern of secreted cytokines and chemokines was qualitatively assessed using cytokine arrays. Supernatant of cells stimulated with SARS-CoV-2 IgG immune complex was tested. The results demonstrate the induction of multiple pro-inflammatory cytokines (**Figure 2A**). For establishment of the experimental system, we focused on the quantitative detection of IL-8, a key player in COVID-19 and neutrophil activation (7, 40–42).

For a comprehensive characterization of our COVID-19 model, we measured phagocytosis and corresponding IL-8 release by treating neutrophil-like HL60 cells with different immune complexes (**Figure 2B**). The data show that phagocytosis and IL-8 release are induced by immune complexes of SARS-CoV-2 spike protein coated beads with specific antibodies against the mentioned spike protein. In controls, the cells display only a very low bead uptake, whereas IL-8 release is not affected. In accordance with the immune complex characterization data (compare **Figure 1C**), cellular inflammation shows no activation with other immunoglobulins.

Next, the previously characterized COVID-19 plasma donations were tested. Phagocytosis of SARS-CoV-2-like immune complex opsonized with plasma from different COVID-19 donors results in varying activation of the cell system (Donor 4 < Donor 1–3 < Donor 6 < Donor 5) (**Figure 2C**). Importantly, phagocytic activity correlates with IL-8 release. When higher levels of immune complex uptake were observed also higher levels of IL-8 release were measured.

To directly show which immunoglobulin classes are functionally relevant for immune complex uptake, phagocytosis after preincubation with FcR blocking antibodies was investigated. SARS-CoV-2-like particles opsonized with COVID-19 plasma immunoglobulin IgG, IgA, and IgM (compare **Figures 1B, C**) exhibited significant decreased particle uptake when FcαRI, FcγRIIA, and combinations of those were blocked. Blocking of FcγRI and FcγRIII did not affect particle phagocytosis. In comparison to blocking of activating FcR, blocking of inhibitory FcγRIIB increases phagocytosis of COVID-19 plasma opsonized particles significantly (**Figure 2E**). Although IgM binding and opsonization of SARS-CoV-2 like particles were detectable (**Figure 1B**), no IgM Fc*µ*R was detectable on HL60 cells (**Figure 2D** and **Figure S4B**, **Table SII**) suggesting no participation in bead phagocytosis.

To compare our neutrophil-like cell model on a functional level with primary cells, we performed phagocytosis assay with primary neutrophils from untouched isolation. As seen for HL60 cells specific phagocytosis of SARS-CoV-2-like immune complex results in inflammatory activation of primary human neutrophils (**Figures S5A, B**). FcR blocking revealed small differences: effect of FcγRIII blocking on phagocytosis was stronger, effect of FcαRI blocking in contrast was lower on primary cells than on HL60 cells (**Figure S5D**).

### Addition of IVIG and Trimodulin Preparation Reduces Inflammation

In patients with severe COVID-19, modulation of the hyperinflammatory immune response is a major goal of therapy (4). IVIG preparations are a fast available therapeutic option in treatment of these severely ill patients (9). The functions of IgA in immunotherapy and especially in IgA and IgM enriched immunoglobulin preparations are poorly studied (43). Therefore, we investigated immune modulation in our COVID-19 cell model by the addition of various concentrations of immunoglobulin preparations. We tested IgG containing IVIG (*IgG Next Generation, Biotest AG*), as well as IgG, IgA, and IgM containing trimodulin (*trimodulin, Biotest AG*). Used lots were tested negative for anti-SARS-CoV-2 neutralizing antibodies. With regard to our characterization data (**Figure 2A**), we measured IL-1ra, IL-10, MCP-1, MIP1α, and IL-8 release as a marker of inflammation.

**Figure 3:**
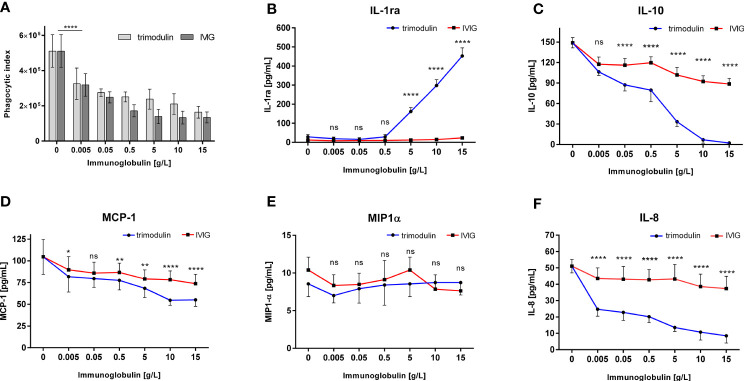
Immune modulation in COVID-19-like model by IVIG and trimodulin preparation. **(A)** HL60 cells were incubated for 1 h with SARS-CoV-2 spike protein coated beads opsonized with COVID-19 plasma. IVIG (*IgG Next Generation*, Biotest AG) or trimodulin (Biotest AG) was added in the indicated concentrations to the cell immune complex mixture. Phagocytosis of SARS-CoV-2-like immune complex was measured with trimodulin (light gray bars) or IVIG (dark gray bars) addition. **(B–F)** Same as **(A)** instead phagocytosis cytokine release into cell culture supernatant was measured with trimodulin (dots, blue line) or IVIG (square, red line) addition. IL1-ra **(B)**, IL-10 **(C)**, MCP-1 **(D)**, MIP-1*α* **(E)**, and IL-8 **(F)** were measured. Values represent mean of six independent experiments. Statistics: Two way ANOVA; Tukey’s multiple comparisons test, 95% confidence interval. ns, not significant, *p ≤ 0.05, **p ≤ 0.01, ****p ≤ 0.0001.

Addition of IVIG or trimodulin to HL60 cells significantly and equally decreased bead uptake of SARS-CoV-2-like particles opsonized with COVID-19 plasma (**Figure 3A**). The corresponding cytokine release is also affected: IL-1ra is strongly induced by trimodulin, not by IVIG addition (**Figure 3B**); IL-10, MCP-1, and IL-8 levels are reduced by IVIG and more significantly by trimodulin addition (**Figures 3C, D, F**). MIP-1α level is not affected by both preparations (**Figure 3E**). The observed effects are dose-dependent. Similar to neutrophil-like HL60 cells, primary neutrophils show reduced phagocytosis and decreased IL-8 release by trimodulin and IVIG addition; however no differences between both preparations were observed (**Figures S5E, F**).

### Trimodulin Induces More Potent ITAMi Signaling Compared to IVIG

The immunomodulatory effects of immunoglobulin preparations are complex and several modes of action work synergistically (20, 44). To better understand the molecular mechanism of immune modulation we tested dependency on inhibitory immunoreceptor tyrosine-based activation motif (ITAMi). The mechanism of ITAMi dependent immune modulation was initially explored for IgA–FcαRI interaction (44, 45) and is a known mode of action for classical IVIG (46). The importance of ITAMi for IgA- and IgM enriched immunoglobulins is so far unknown.

**Figure 4:**
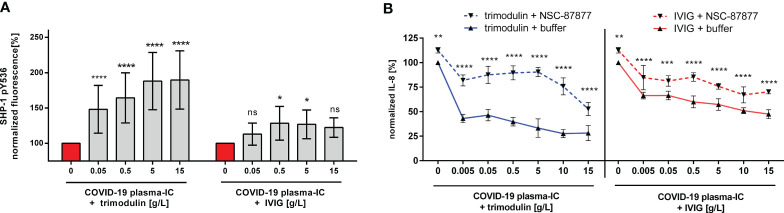
Stronger immune modulation by trimodulin due to more potent ITAMi signaling. **(A)** Phosphorylation of SHP-1 at tyrosine 536 (pY536). Cells were treated for 1 h with COVID-19 plasma-IC and indicated trimodulin or IVIG concentrations for 90 min. Cells were fixed, permeabilized, and stained with anti-phospho SHP-1 pY536 antibody. Fluorescence signal was measured by flow cytometry and normalized to buffer treated cells. **(B)** Blocking of SHP-1 phosphatase reduces immune modulatory effects. Cells were treated as in **(A)**, but additional pre-incubation with 200 µM SHP-1 inhibitor NSC-87877. IL-8 release was normalized and compared between NSC-87877 (dotted lines) or buffer treatment (solid lines). Incubation of cells with trimodulin (blue lines) or IVIG (red lines) is shown with or without SHP-1 inhibitor. Values represent mean of six independent experiments. Statistics: Two way ANOVA; Tukey’s multiple comparisons test, 95% confidence interval. ns, not significant, *p ≤ 0.05, **p ≤ 0.01, ***p ≤ 0.001, ****p ≤ 0.0001

To directly demonstrate ITAMi activation due to trimodulin or IVIG addition, we measured phoshorylation level of SHP-1. As seen in **Figure 4A** trimodulin induces significant stronger phosphorylation of SHP-1 than IVIG. Furthermore, inhibition of SHP-1 phosphatase reduced significantly the strong immune modulatory effects of trimodulin which were observed on IL-8 release. Similar effects but to a lower extent were seen by IVIG addition (**Figure 4B**).

## Discussion

The rapid global spread of SARS-CoV-2 virus induced COVID-19 disease leads to an urgent need for appropriate therapeutics. The major issue for severe COVID-19 patients is a dysregulated immune system with hyperinflammation, cytokine storm, ARDS, and ultimately respiratory failure (4, 12, 47). Previous data indicate that in severe cases excessive immune response, mediated by neutrophils (35), is of greater importance for COVID-19 progression than virus induced damage alone, highlighting the importance of immune modulators (9). Besides clinical studies investigating the efficiency of immune modulators, there is an urgent need for powerful models estimating the immune modulatory potency and to unravel modes of action of potential therapeutics. Because severe COVID-19 cannot yet be depicted in animal models (48), we developed an *in vitro* model to fill this gap.

Neutrophils are well suited as a model system in the context of COVID-19 for several reasons: (1) neutrophils have an elementary role in the human innate and adaptive immune systems, and (2) they mediate antibody mediated effector functions which play a central role in COVID-19 disease severity (26, 35, 49). (3) Neutrophils are poorly studied in conjunction with viral respiratory diseases (40) (4). *In vivo* studies using mice in the context of neutrophil FcR functions are limited because of crucial differences between mice and humans: neutrophil abundance in mice is lower; neutrophil related chemokines like IL-8 are missing. Furthermore, Fc*α*RI, Fc*γ*RIIA, and Fc*γ*RIIC are not expressed in mice, and IgG as well as IgA antibody classes differ (24, 50).

As a basis to study immunomodulation at the cellular level, as an easy-to-use and versatile platform to activate pro-inflammatory immune cells, we chose a bead platform with fluorescent latex beads (51–53). Our bead system has the advantage to easily monitor inflammatory and anti-inflammatory processes under standard laboratory conditions. To associate the bead system with COVID-19, we coated the beads with recombinant full-length SARS-CoV-2 spike protein. The SARS-CoV-2 spike protein was selected because it is responsible for virus infectivity and mediates antibody-dependent immune response against the virus *in vivo* (8, 54). With this model system, it is possible to unravel, in detail, immunomodulatory mode of actions by immunoglobulin preparations that are so far unknown in the context of COVID-19. A specific focus of our work is to show the importance of the neglected IgA antibodies in immunotherapy.

### SARS-CoV-2-Like Immune-Complex Were Generated by Specific IgG, IgA, and IgM Antibodies

Besides IgG, neutralizing antibodies from IgA and IgM classes are important, especially in the early immune response. Nevertheless, they were often not monitored in patients (28, 55–59). It was shown, that disease severity is linked to anti-SARS-CoV-2 spike protein specific antibody levels, corresponding to FcR mediated antibody functions and inflammatory cytokine release (8, 26, 60). To understand the mechanism of SARS-CoV-2-like particle uptake *via* immunoglobulin opsonization, IgG, IgA, and IgM binding on the particle surface were detected.

It was shown that the convalescent COVID-19 plasma opsonized beads showed binding of IgG, IgA, and IgM classes to the SARS-CoV-2 spike protein on the bead. This is in accordance with ELISA data showing antibodies of IgG, IgA, and IgM classes against SARS-CoV-2 surface proteins (55, 58, 60).

Furthermore, we tested six different donors from an unknown time point of plasma donation after infection. The varying levels of IgM, IgA, and IgG antibodies can be explained by the immune response which changes from IgM to IgG and IgA in the latter phase (60–62). In particular, neutralizing IgM levels, but also IgA and IgG levels, decline several weeks after infection (61, 62).

### Fc*γ*RIIA- and Fc*α*RI-Dependent Phagocytosis Induces COVID-19-Like Inflammation

The basis for COVID-19-like inflammation in our *in vitro* model is FcR-dependent phagocytosis of SARS-CoV-2-like immune complex by immune effector cells. As effector cells, we use the well characterized HL60 cell line, which is used for a variety of phagocytosis assays (36, 63). To ensure that the differentiated HL60 cell line has the desired neutrophil-like phenotype, surface marker and FcR expression were analyzed. Neutrophil-like HL60 cells were compared with primary human neutrophils, as well as non-differentiated HL60 cells. Typical neutrophil phenotype markers for this cell-line and FcR expression pattern changed during differentiation to a neutrophil-like phenotype, as reported by others (36, 63–67). ACE-II receptor expression was not reported to be present on circulating immune cells (42, 68). Similarly, we found negligible ACE-II expression on HL60 neutrophil-like cells and primary neutrophils. Based on this knowledge antibody Fc-part mediated functions of IgG and IgA antibody class can be investigated without effects by ACE-II receptor.

Stimulation of neutrophil-like HL60 cells or primary human neutrophils with SARS-CoV-2-like immune complex resulted in secretion of several pro-inflammatory chemokines and cytokines like MCP-1, MIP-1α, IL-1β, IL-8, IL1ra, or IL-10. Upregulation of these inflammatory mediators was also observed in patients with severe COVID-19 [reviewed in (69)]. For more detailed and mechanistic investigations of our model we decided to monitor IL-8. Increase in IL-8 level has been reported in COVID-19 patients by various studies (5, 70, 71). The phagocytosis of SARS-CoV-2-like immune complex was linked to a reproducible increase in IL-8 release by neutrophil-like HL60 and primary cells. Observed differences in phagocytic activity and cytokine profile of primary neutrophils and neutrophil-like HL60 cells arise mainly due to enhanced phagocytic potency and short half-life of primary cells as reported (63, 72–74). This data justifies the suitability of our model system to depict severe COVID-19 and the usage of HL60 cells as alternative to primary neutrophils.

COVID-19 convalescent plasma was heat inactivated to inhibit classical or alternative complement pathway (75); therefore the assay focuses on antibody-dependent mechanism.

As shown by others, the antibody dependent neutralization of SARS-CoV-2 correlates with specific IgG, IgA, and IgM antibody levels against viral surface proteins (76). In accordance with the IgG, IgA, and IgM levels detected on the bead surface we found varying phagocytosis of immune complex generated with plasma from different donors. With these tested donations as example, we showed diversity of donations (with specific IgG, IgA, and IgM antibodies) and the implications for the established assay.

Noteworthy, is the correlation between antibody distribution (IgG, IgA, or IgM) and level of phagocytosis. Donors with high IgM levels showed lower phagocytosis and IL-8 release than donors with the same IgA or IgG but lower IgM. As only antibodies from IgG or IgA class can mediate antibody-dependent phagocytosis *via* their FcR (77), using neutrophils with no IgM Fc*µ*R (78) (as well as on the here used neutrophil-like HL60 cells) and inactivation of complement, the assay setup can explain this effect.

The reliance on specific immunoglobulins for particle uptake indicates FcR dependency, as reported for SARS-CoV by others (79, 80). The phagocytosis of COVID-19 plasma opsonized particles was dependent on FcαRI, FcγRIIA or combinations of both. This dependency shows that IgA and IgG are functionally relevant in this complement-free setup. No influence on particle uptake was detected for FcγRIII. This could be due to lower expression in neutrophil-like HL60 cells compared to primary neutrophils. FcR blocking on primary cells revealed that even though the highly expressed FcγRIII is part of immune complex phagocytosis, the lower expressed of FcγRIIA is functionally of greater importance (81, 82). Furthermore, neutrophils of severe COVID-19 patients exhibit lower levels of FcγRIII (83–85). This highlights the suitability of neutrophil-like HL60 cells as a model system for COVID-19. The observed effects are known for Fc-receptors in relation to immunoreceptor tyrosine-based activation motif (ITAM) and is mediated *via* Fc-receptor ITAM-signaling (44, 86). The contrary effects, shown by blocking FcγRIIB, are known to correspond to inhibitory immunoreceptor tyrosine-based inhibition motif (ITIM) (81, 86). This was also demonstrated for the here established model (data not shown).

To summarize the results, we demonstrate high specificity and low background signal, making this assay a powerful platform to study virus and bacterial induced inflammation. The *in vitro* data from several donors show that immunoglobulin classes IgG and IgA are important for antibody-dependent phagocytosis of SARS-CoV-2 virus-like particles. This is consistent with clinical data showing antibody response from IgG and IgA type against SARS-CoV-2. Although, again underestimated, IgA seems to have an important role, especially in the early immune response (55–58).

### Trimodulin Exhibits Strong Immune Modulation *via* ITAMi Signaling

As described above, patients with severe COVID-19 could benefit from immune modulators (4). Immunoglobulin preparations are a fast available and long used therapeutic option in treatment of inflammatory diseases (9). The role of IgA in IgA and IgM enriched immunoglobulin preparations is fairly unknown or neglected (22, 43). To shed light on IgA we compared IgG (~98%) containing IVIG (*IgG Next Generation, Biotest AG*), as well as IgG (~56%), IgA (~21%), and IgM (~23%) containing trimodulin (*trimodulin, Biotest AG*), regarding their anti-inflammatory effects in our COVID-19 cell model. Trimodulin was shown to be effective in ventilated patients with severe community acquired pneumonia and high inflammation markers (CIGMA-Study) (87) and is therefore in clinical testing for related COVID-19 disease (19). The lots of these products were manufactured from plasma collected before COVID-19 pandemic and had no anti-SARS-CoV-2 neutralizing antibodies (*in house data*) (88).

Addition of therapeutically used concentrations IVIG (89, 90) or trimodulin similarly block the binding and uptake of SARS-CoV-2-like immune-complex by FcR, as seen by subsequent reduction of phagocytosis. Inhibition of phagocytosis due to trimodulin and IVIG treatment impairs viral clearance. Nevertheless, this is not detrimental because treatment is limited to patients with severe COVID-19 where viral load is of minor importance, and modulation of the exhausted immune system is the major goal of therapy (10).

However, the corresponding effector outcome (cytokine release) shows clearly a stronger immune modulation by trimodulin, which was shown to be in part FcαRI-dependent. Based on our characterization data and COVID-19 literature, we observe modulation of cytokine release as indicated by increased levels of anti-inflammatory and decreased levels of pro-inflammatory cytokines (5–7). The reductions of pro-inflammatory cytokines like IL-8 and MCP-1 are commonly known aims of severe COVID-19 therapy (6, 12, 91). Additionally, the blockade of inflammatory IL-1 pathways with IL-1 receptor antagonists is a promising therapeutic option (92). The strong upregulation of IL-1ra by trimodulin treatment could lead to similar effects. IL-10 is described in most articles as an anti-inflammatory cytokine; however, this role is currently under discussion (93). COVID-19 patients show elevated IL-10 levels which were known to correlate with disease severity and mortality (94, 95). Increasing evidence suggests IL-10 as promotor of inflammation similar to IL-6 (93, 96, 97). The reduction of IL-10 levels due to trimodulin treatment could therefore be beneficial for severe COVID-19 patients.

Immunomodulation was observed with HL60 cell model and primary human neutrophils treated with trimodulin or IVIG. In contrast to the cell-line model, primary cells show comparable effects of trimodulin and IVIG. This could be due to: first, primary neutrophils express more IgG FcR, especially FcγRIII, in relation to IgA FcαRI. Second, HL60 cell lines are tumor cells with a more inflammatory basal phenotype than neutrophils directly isolated from healthy donors (36). For FcαRI increased expression and enhanced avidity between FcαRI and IgA are known as “inside-out” signaling for inflammatory environment (98–102). The more powerful immunomodulation elicited by trimodulin is therefore especially relevant in inflammatory environment (like in COVID-19 patients) when the additional IgA component can interact more strongly with its receptor Fc*α*RI.

The modulation of the immune response by IVIG is hypothesized to be a combination of several modes of action. Blocking of activating FcR, targeting of inhibitory FcR-signaling, modulation of FcR-expression, and the interaction with soluble inflammatory molecules (complement factors, cytokines) are ways how immunoglobulins modulate the immune response (44, 103, 104). Similarly, in this *in vitro* setup, it is likely that several modes of action work in combination.

Induction of inhibitory ITAM (ITAMi) signaling *via* IgG–Fc*γ*RIIA-axis is a known IVIG mode of action (46, 105). ITAMi-maintained immune homeostatic conditions by counteracting ITAM induced effector outcomes, like reduced reactive oxygen species (ROS) or inflammatory cytokine release (44, 45). Further, immune modulation *via* monovalent targeting of IgA–Fc*α*RI-axis is known for several *in vitro* and *in vivo* models (31, 45, 106–108). Here we show for the first time immune modulation by IgA and IgM enriched immunoglobulin trimodulin *via* ITAMi signaling. Trimodulin induces a more powerful ITAMi than IVIG resulting in stronger immune modulation. Based on experimental design, the role of trimodulin IgM component is of minor importance for FcR mediated effector functions; beneficial immunomodulatory effects (in comparison to IVIG) can therefore be attributed to the IgA component. Explanation for this superior ITAMi signaling could be the 2:1 stoichiometry by IgA–FcαRI binding, whereas IgG binds FcγR in 1:1 stoichiometry (109, 110). The data from this work substantiate the theory of IgA as immune modulator in COVID-19 therapy (43, 111).

Comments to first clinical trials of high dose IVIG treatment in COVID-19 patients mention these modes of action (112, 113), but this work gave the first *in vitro* data confirming this hypothesis. Whether the immunomodulatory treatment with trimodulin is beneficial for severe COVID-19 patients is under evaluation in clinical study (19). A first case report using Pentaglobin^®^, another IgA- and IgM enriched immunoglobulin preparation, showed decreased inflammatory markers (18), confirming the here depicted hypothesis of IgA containing immunoglobulins as immune modulator. Monitoring of IgA antibody response in COVID-19 patients is neglected in many studies although especially in early weeks after SARS-CoV-2 infection and in severe cases the IgA response seems to be important (55–58, 70, 114). By screening patients with strong IgA mediated inflammatory response particularly trimodulin, therapy could be promising.

Other threats for severe COVID-19 patients are co-infections, which were shown to worsen clinical outcome. Various studies highlight that a relevant portion of COVID-19 patients suffer from bacterial or viral co-infections (115–118). Immunoglobulin preparations combine several modes of action and could therefore perform immune modulatory functions while simultaneously fighting co-infections (9, 119). Anti-microbial effects of trimodulin, as well as reduction of secondary bacterial infections in severe patients, are hints that trimodulin could improve clinical outcome of co-infected COVID-19 patients (87, 120).

However, this study has some limitations; first, the number of donors used for evaluation of assay performance is limited, and no information regarding time point of plasma donation, sex, age, or disease severity is available. Further studies could focus on plasma donations and the different effector functions of SARS-CoV-2 specific antibodies of IgG, IgA, and IgM class. Another limitation is that we investigate immunomodulatory properties of IVIG and trimodulin solely with neutrophils and a limited number of cytokines as indicators for hyperinflammation. Whether other immune cells, like monocytes, macrophages, or NK-cells are important targets of IVIG therapy, will be evaluated in further studies.

### Concluding Remarks

It is important to note that the whole immune system is modified and exhausted in severe COVID-19 patients, with both the adaptive and innate immune systems affected (9, 121). The here developed neutrophil *in vitro* model is an artificial system and cannot depict this complexity. It is rather one instrument to understand processes in the fight against COVID-19. Besides COVID-19, the inflammation model can be easily adapted to every pathogen or immune cell of interest, making it a versatile platform for immunological investigations.

The here demonstrated immunomodulation induced by immunoglobulin preparations could be beneficial for the treatment of COVID-19 induced hyperinflammation. The data indicate stronger modulation of pro- and anti-inflammatory cytokines by trimodulin in comparison to standard IVIG treatment. Advantages could be shown to be due to the additional IgA component and ITAMi signaling. Particularly in focus of respiratory diseases, like COVID-19, IgA could be an important therapeutic molecule for immune modulation. The observed immune modulatory effects of trimodulin have yet to be tested in clinical studies to demonstrate the efficacy of this product class.

## Data Availability Statement

The original contributions presented in the study are included in the article/**Supplementary Material**. Further inquiries can be directed to the corresponding author.

## Author Contributions

FB and SF designed experiments. FB performed experiments. FB wrote the manuscript. SF designed figures. SF, SW, DR, MG, and JS reviewed and edited the manuscript. All authors contributed to the article and approved the submitted version.

## Funding

Biotest AG gave financial support for the research conducted in this study.

## Conflict of Interest

All authors are employees of Biotest AG, Dreieich, Germany.

The authors declare that this study received funding from Biotest. The funder had the following involvement in the study: study design, interpretation of data, the writing of this article and the decision to submit it for publication.
