# Supplementary material for: Immunomodulation: Immunoglobulin Preparations Suppress Hyperinflammation in a COVID-19 Model via FcγRIIA and FcαRI
Source: Front Immunol. 2021 Jun 10;12:700429. doi: 10.3389/fimmu.2021.700429 (PMC8223875; doi:10.3389/fimmu.2021.700429)
Supplement: Supplementary file 1 [file DataSheet_1.docx]

Supplementary Material

# Supplementary Data

## Supplementary tables

Table SI: Antibodies used in flow cytometry for immunological staining of primary human neutrophils and HL60 neutrophil-like cell surface markers and Fc-receptors.

| **Antigen** | **Mouse anti-human clone** | **Fluorophore** | **Manufacturer #Art. no.** | **Used volume** |
| --- | --- | --- | --- | --- |
| CD15 | W6D3 | PE | BD Biosciences #562371 | 5 µL |
| CD11b | ICRF44 | AF488 | StemCell #60040AD | 5 µL |
| CD35 | E11 | AF647 | BD Biosciences #565329 | 5 µL |
| CD71 | M-A712 | APC-H7 | BD Biosciences #563671 | 5 µL |
| CD193 | 5E8 | BV421 | BD Biosciences #562570 | 5 µL |
| ACE-II | E-11 | PE | SantaCruz Biotech #sc390851-PE | 1 µL |
| FcγRI | 10.1 | PE-Cy7 | BD Biosciences #561191 | 5 µL |
| FcγRIIA | IV.3 | FITC | StemCell #60012FI | 20 µL |
| FcγRIIB | 2B6 | AF647 | Creative BioLabs #TAB-036WM | 2.5 µL |
| FcγRIII | 3G8 | APC-Cy7 | BD Biosciences #557758 | 5 µL |
| FcαRI | A59 | BV421 | BD Biosciences #744374 | 3 µL |
| FcµR | HM14-1 | BV421 | BD Biosciences #564714 | 5 µL |

Table SII: Comparison of cell surface marker and Fc-receptor expression on not differentiated HL60 cells, differentiated HL60 cells (+1.3% DMSO, 4 days) and primary neutrophils isolated from human blood donations. The percentage of positive cells for the indicated surface protein from single cell gates are depicted. Data represent mean percentage of 6 independent experiments (in case of primary neutrophils, 6 different donors).

| Surface protein | **HL60 cells**  **[% positive cells]** | **HL60 cells + 1.3% DMSO 4 days**  **[% positive cells]** | **primary neutrophils**  **[% positive cells]** |
| --- | --- | --- | --- |
| CD15 | 100,0 | 100,0 | 100,0 |
| CD35 | 50,1 | 91,9 | 99,6 |
| CD11b | 5,9 | 73,2 | 99,8 |
| CD71 | 76,5 | 19,5 | 0,4 |
| CD193 | 7,1 | 0,4 | 0,2 |
| ACE-II | 7,4 | 14,6 | 12,5 |
| FcγRI | 81,4 | 92,7 | 35,1 |
| FcγRIIA | 95,2 | 98,0 | 99,7 |
| FcγRIIB | 15,0 | 9,3 | 9,9 |
| FcγRIII | 3,1 | 29,1 | 99,7 |
| FcαRI | 25,1 | 95,8 | 99,9 |
| FcµR | 8,2 | 4,2 | 9,3 |

## Supplementary figures


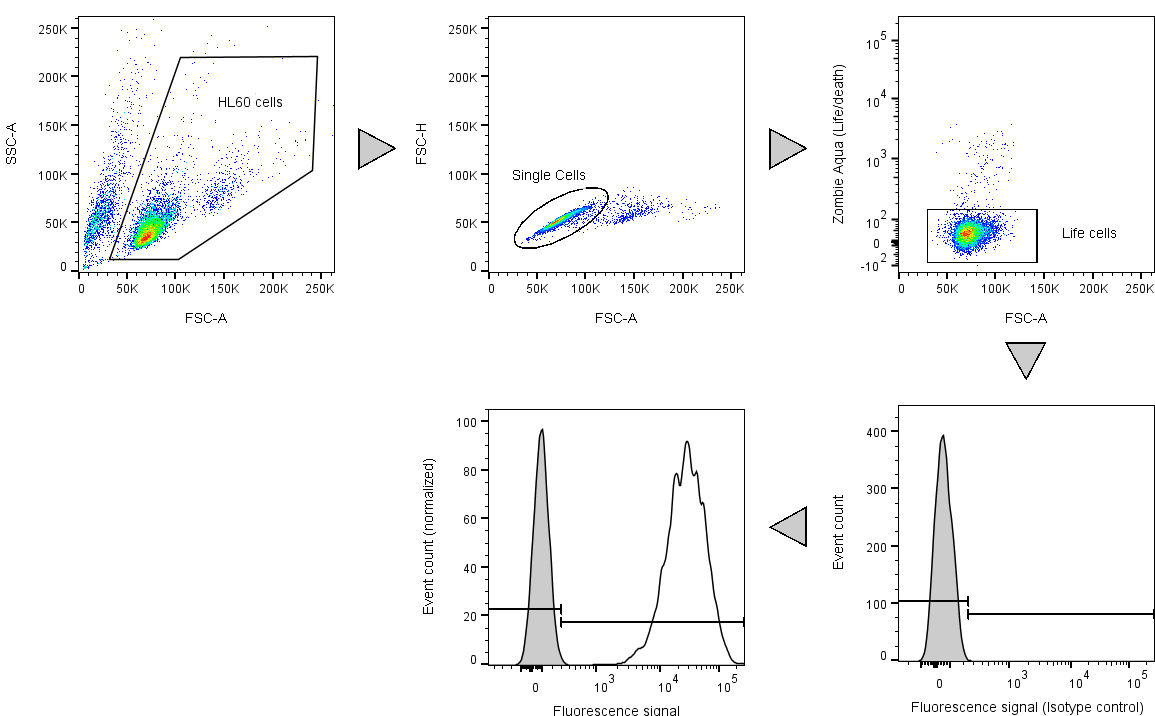


***Figure S1:*** Gating strategy of HL60 cells and primary neutrophils for surface marker staining. Cell population was gated based on SSC/FSC to exclude debris, followed by single cell gating. To exclude death cells viability staining using Zombie-Aqua (BioLegend) was performed. Threshold gate for each fluorescence channel was set using isotype controls. Isotype controls are shown as grey filled histograms, stained cells are shown as black histograms. Data show representative values from 6 independent experiments.


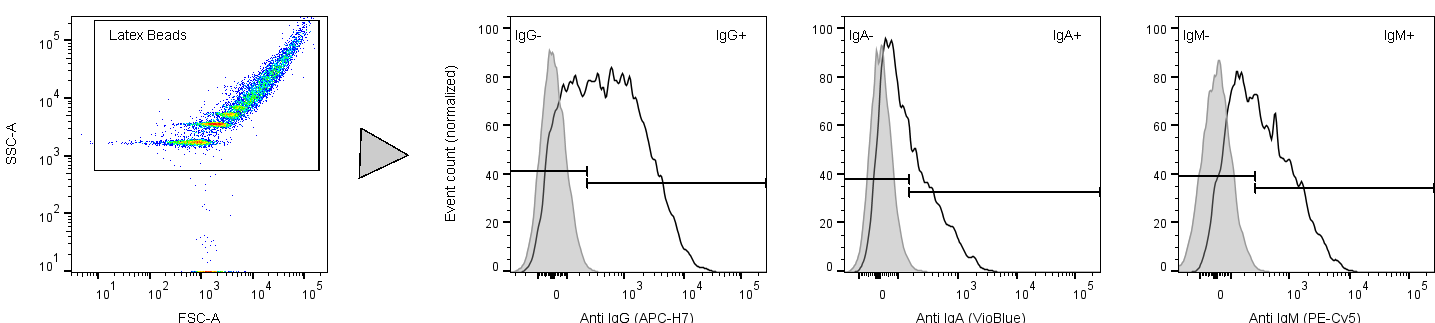


***Figure S2:*** Gating strategy for detection of IgG, IgA and IgM on SARS-CoV-2 spike protein coated latex beads opsonized with immunoglobulins from COVID-19 plasma. Latex bead population was gated on logarithmic FSC-SSC values, gates for IgG, IgA and IgM positive beads were set based on isotype controls. Isotype controls are shown in grey filled histograms; stained bead-populations are shown as black histograms. Data show representative values from 6 independent experiments.


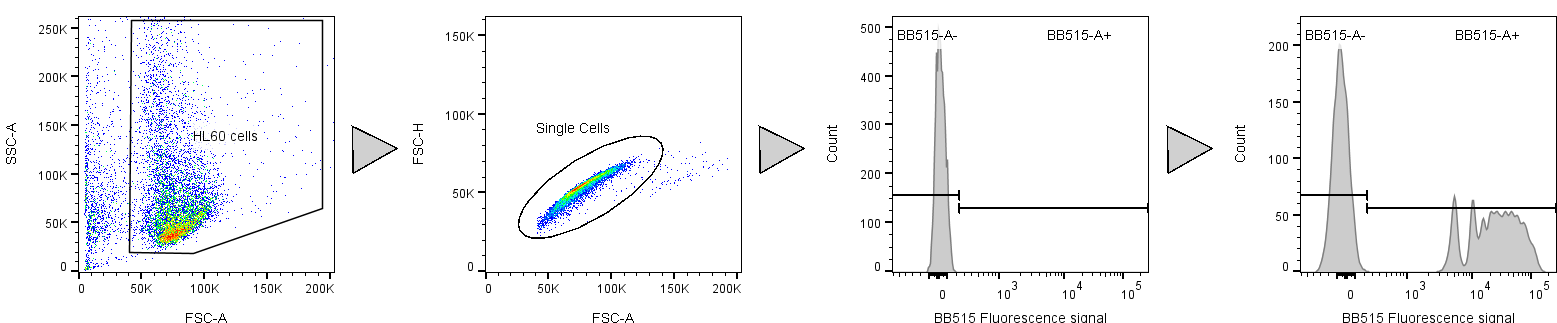


***Figure S3:*** Gating strategy of phagocytosis assay. Cell population was gated based on SSC/FSC to exclude debris, and gate single cells, followed by setting a threshold gate for BB515 positive cells based on untreated cells. The percentage and MFI of BB515 positive cells were analyzed and multiplied to calculate phagocytic index. Data show representative values from 8 independent experiments.


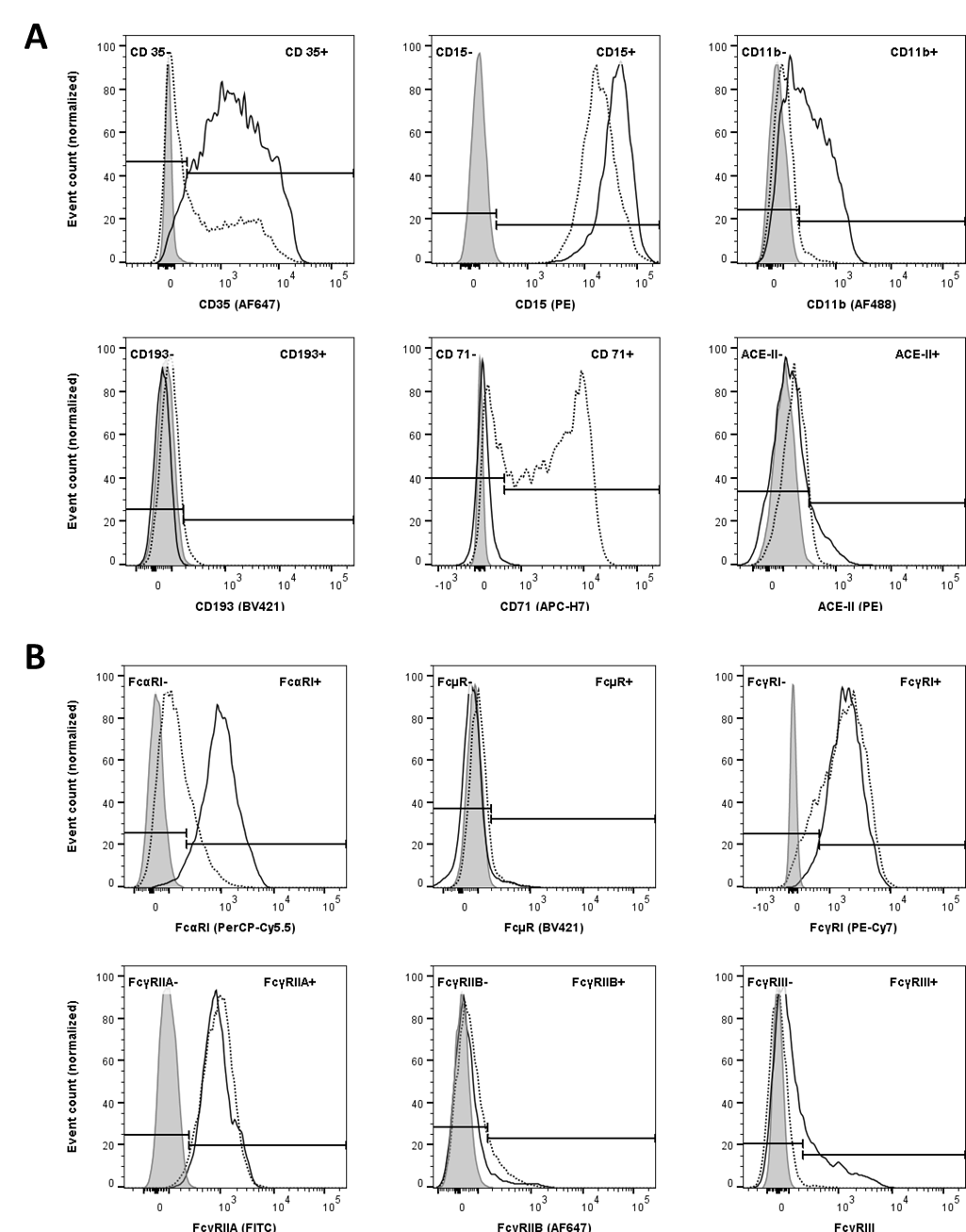


***Figure S4:*** Comparison of HL60 neutrophil-like phenotype after 4 days differentiation with not differentiated HL60 cells by flow cytometry. **(A)** Expression of neutrophil differentiation markers CD35, CD15, CD11b, CD193 and CD71. As well as SARS-CoV-2 receptor ACE-II. **(B)** Expression of IgG (FcγRI, FcγRIIA, FcγRIIB, FcγRIII), IgA (FcαRI) and IgM (FcµR) FcR. HL60 cells, 4 days differentiated with 1.3% DMSO were harvested, washed and stained with specific detection antibodies of indicated surface proteins and analyzed in FACS Canto II cytometer. Isotype controls were depicted in gray filled histogram, histograms of differentiated cells with black solid lines, histograms of not differentiated cells in dotted lines. Data show representative values of 6 independent experiments


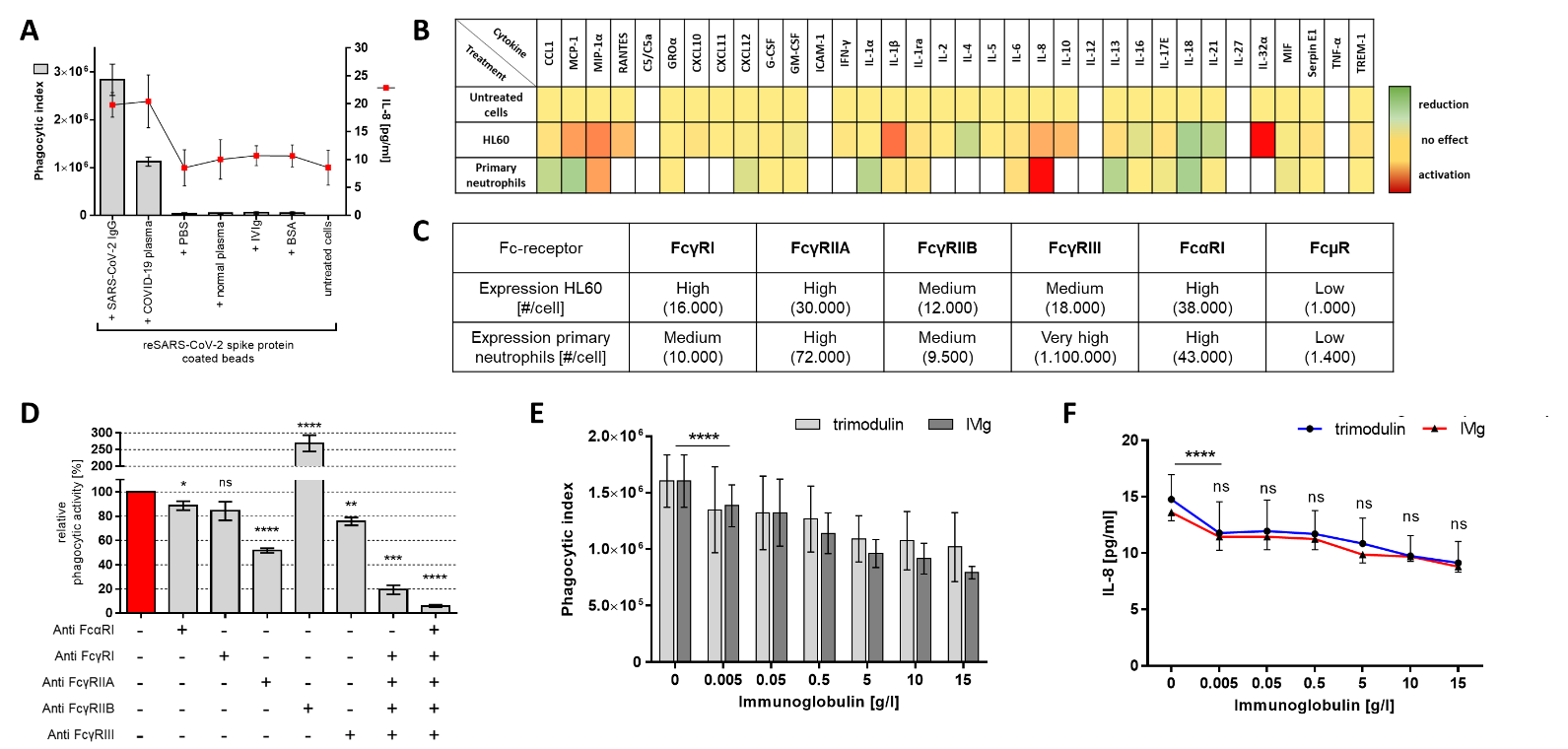


***Figure S5:*** COVID-19 inflammation model with primary human neutrophils. **(A)** Evaluation of COVID-19-like inflammation model. Cells were incubated with SARS-CoV-2 spike protein coated beads opsonized with different immunoglobulins, BSA or PBS controls. Phagocytic index (gray bars, left y-axis) for bead uptake and corresponding IL-8 release (red dots, right y-axis) in cell culture supernatant was measured. Data represent mean of 3 donors. **(B)** Heat map of chemokines and cytokines secreted by neutrophil-like HL60 cells or primary neutrophils after stimulation with anti-SARS-CoV-2 IgG-IC. Cells were stimulated for 18 h at 37° C with the immune-complex. Qualitative cytokine secretion was measured by using human cytokine arrays. Relative signal intensities for every measured cytokine of untreated cells were set as 1 (yellow). The x-fold induction (red) or reduction (green) in comparison to untreated cells was calculated. Not detected cytokines are shown in white. Results represent mean of 3 independent experiments. **(C)** Comparison of human Fc-receptors (FcR), immunoglobulin binding specificity and their expression number [#/cell] on neutrophil-like HL60 cells or primary neutrophils. Fc-receptor quantification on HL60 cells, 4 days differentiated with 1.3% DMSO, or primary neutrophils isolated from human blood was performed using QuickCal calibration beads, results represent mean of 6 independent experiments. **(D)** FcR blocking experiments with COVID-19 plasma opsonized SARS-CoV-2-like particles. Cells were pre-incubated with 5 µg/mL of indicated blocking antibodies or combinations of those 20 min before addition of immune-complex. Phagocytic index of not blocked cells were referred as 100% phagocytic activity and remaining phagocytic activity is shown as mean of 4 independent experiments. **(E)** HL60 cells were incubated with SARS-CoV-2 spike protein coated beads opsonized with COVID-19 plasma. IVIg (*IgG Next Generation*, Biotest AG) or trimodulin (Biotest AG) were added in the indicated concentrations to the cell immune-complex mixture. Phagocytosis of SARS-CoV-2-like immune-complex was measured with trimodulin (light gray bars) or IVIg (dark gray bars) addition. **(F)** Same as (E) instead phagocytosis cytokine release into cell culture supernatant was measured with trimodulin (dots, blue line) or IVIg (triangle, red line) addition. Statistics: One way ANOVA; Dunetts multiple comparisons test, p ≤ 0.1 *, p ≤ 0.01 **, p ≤ 0.001 ***, p ≤ 0.0001 ****, 95% confidence interval.

## Supplementary methods

### Isolation of primary human neutrophils

Primary human neutrophils were isolated by immunomagnetic depletion using MACSxpress® Whole Blood Neutrophil Isolation Kit (Miltenyi Biotec). Isolation was performed according to manufactures instructions. In short human blood donations were mixed with magnetic beads coupled with antibody cocktail to deplete non target cells. Neutrophils were separated from non target cells using magnetic separator and erythrocytes were sedimented by aggregation. Supernatant was removed, centrifuged and remaining red blood cells lysed with red blood cell lysis buffer (Thermo Fisher Scientific).

### Phagocytosis assay with primary human neutrophils

Phagocytosis of SARS-CoV-2-like immune complex was performed as described for HL60 cell line. Divergent primary neutrophils were rest for minimum of 1 h after isolation in Roswell Park Memorial Institute medium (RPMI) with 5% FBS (1). Centrifugation steps were performed at 250 x g, 5 min and phagocytosis was performed in RPMI medium with 5% FBS for 1 h.

### FcR Quantification

Quantification of FcR on HL60 neutrophil-like cells and primary neutrophils was performed as described previously (2) using Quantum Simply Cellular Beads (Bangs Laboratories Ltd.). FcR number per cell was determined by calculating the antibody binding capacity (ABC) based on a reference curve with beads of known antibody binding capacity. The median fluorescence intensity of each FcR staining was compared to the median fluorescence intensity of the calibration curve and ABC value was calculated.
